# Optimization of cultivation techniques improves the agronomic behavior of *Agaricus subrufescens*

**DOI:** 10.1038/s41598-020-65081-2

**Published:** 2020-05-18

**Authors:** Arturo Pardo-Giménez, José Emilio Pardo, Eustáquio Souza Dias, Danny Lee Rinker, Cinthia Elen Cardoso Caitano, Diego Cunha Zied

**Affiliations:** 1 Centro de Investigación, Experimentación y Servicios del Champiñón (CIES), Quintanar del Rey, Spain; 2 Escuela Técnica Superior de Ingenieros Agrónomos, Universidad de Castilla-La Mancha, Albacete, Spain; 3 Universidade Federal de Lavras, Departamento de Biologia, Lavras, Brazil; 4 University of Guelph, Vineland Campus, VinelandStation, Guelph, Canada; 5 Universidade Estadual Paulista (UNESP), Faculdade de Ciências Agrárias e Tecnológicas (FCAT), Dracena, Brazil

**Keywords:** Microbiology techniques, Fungi

## Abstract

New species of medicinal mushrooms have emerged over the past several decades, such as the Sun mushroom, *Agaricus subrufescens*. Horticultural improvements are required to shift its cultivation from small-scale local production to large-scale international production. The research reported here evaluated the agronomic behavior and the chemical characteristics of the Sun mushroom as a function of i) nutritional supplementation ii) ruffling of the casing layer and iii) the temperature management on the primordia induction and reduction of the crop cycle. Supplementation was beneficial for yield, unit mushroom weigh and decrease in time to first harvest. Supplementation improved biological efficiency with Champfood providing a yield increase of 15% over the non-supplemented compost. Among the supplements only Promycel increased the individual mushroom weight. Ruffling overall improved the yield in the 2^nd^ and 4^th^ flush. Already biological efficiency was greater by 21%. The highest yield harvested in any single day in the crop occurred in 3^rd^ flush with the amount of 2.484 kg of mushrooms per m^2^ for the rapid induction method. Still the biological efficiency was not significantly affected by the mushroom induction temperature method. Only the fat content of the mushrooms was positively affected by the rapid induction of primordia. Champfood supplement promotes a reduction in the value of earliness and an increase of 1^st^ flush yield. The ruffling technique provided an increase in biological efficiency due to the great number of mushrooms harvested. Rapid primordia induction allowed the crop cycle to end 3 days earlier than the slow primordia induction, providing a higher production rate.

## Introduction

New species of medicinal mushrooms have emerged over the past decades. One such mushroom is the Sun mushroom, reported as *Agaricus subrufescens* Peck, *Agaricus brasiliensis* Wasser et al. and *Agaricus blazei* (Murrill) ss. Heinemann^1–4^. This species has been cultivated on a small-farm scale in Brazil for many years.

In 2017 Royse *et al*.^5^ reported that world mushroom production is divided among several genera: *Lentinula* (22%), *Pleurotus* (19%), *Auricularia* (18%), *Agaricus* (15%), *Flammulina* (11%), *Volvariella* (5%) and others (10%). In the discussion of *Agaricus* these authors did not mention the Sun mushroom. In 2018 Sanchez *et al*.^6^ noted that the only country in the Americas that cultivated the Sun mushroom was Brazil. *Agaricus subrufescens* along with *Pleurotus eryngii*, *Flammulina velutipes* and other non-Agaricus represented only 6% of the total production in Brazil.

Changes in horticultural technologies are required to shift production from small-scale local producers to large-scale commercial production with international markets. Two main challenges in the cultivation of Sun mushroom are the compost nutrition and its long cultivation cycle^7,8^.

Production of *A. subrufescens* generally follows that of *Agsricus bisporus*. Despite this, the cultivation of the button mushroom has a much shorter cultivation cycle and a much higher yield. Therefore, the two species can be grown using similar technology, however, the results are not the same. In *A. bisporus* cultivation, hybrid strains, are grown preferentially, because of the high quality and yield of the fruit-bodies. An important advance in this area occurred with the development of the well-known Horst^®^ U-strains, on crossing white strains with off-white strains^9^. The spawn cultures used in *A. subrufescens* cultivation are from wild strains, which have not yet undergone a breeding process, as has already been the case with the main cultivated mushrooms species^10^. Llarena-Hernández *et al*.^11^ analyzed European wild strains, which showed agronomic parameters superior to those of Brazilian strains. Two strains showed similar production to the button mushroom, however, after a long cultivation cycle. In addition, these strains showed greater difficulty in colonizing the compost. Another important observation was that a same strain showed a wide variation in productivity between different experiments^12^. Then, the cultivation of the almond mushroom is still complex and even though some hybrids have been tested, no commercial company offers spawn for growers. Nowadays, strains are circulating through private collections and are generally not widely available^13^. Moreover, Wisitrassameewong *et al*.^3^ did not exclude the fact that *A. subrufescens* might be a complex of species. Therefore, given all the above considerations, there is no doubt that there is still a long way to go to make the cultivation of this mushroom as efficient as was achieved for the button mushroom.

The almond mushroom is cultivated on farms where there are multiple crops at different stages in protected structures. Raw materials consist typically of a cereal straw (eg., wheat, barley, rice) that provide carbohydrate and lignin. These bulk materials are low in nitrogen and the compost is enriched through animal litter (eg., chicken or turkey manure) and other available nitrogen sources. Gypsum (CaSO_4_) is added to conserve nitrogen, balance the pH and serve as a flocculant. These heterogenous materials are mixed, hydrated and composted for 2 to 3 weeks. After this period, the compost is pasteurized and further composted under strict environmental controls. The composting process is stopped once the ammonia has disappeared. At this time this substrate may be supplemented with additional nutrient products (as per the research in this paper) by thoroughly incorporation. Spawn (mycelia of the fungus colonized on sterile grain) is mixed into this substrate. For 2 to 3 weeks the mycelia colonize the substrate (a.k.a. spawn run). At the end of this growth, the substrate may be supplemented as well. Then, the surface of the colonized substrate is top-dressed with a layer of material(s) (a.k.a. casing layer) buffered with lime that aids in the induction of the mushroom primordia. Within 7 to 15 days mushroom formation (a.k.a. primordia induction or initiation) is initialized through changes in substrate and air temperatures and gaseous management. Once the mushrooms form and are market ready, they are harvested by hand. Mushrooms grow in cycles (a.k.a. flushes). The crop is terminated after a set period time that has been determined to be economically viable.

Commercial cultivation of *A. bisporus* utilizes compost supplementation, a ruffling of the casing layer (mixing of mycelium and materials *in situ*), and various schema of temperature manipulation for primordia initiation. Supplementation is the incorporation of high nitrogen materials into the compost at spawning or casing to improve yield^14^. Compost supplementation provides nutrients to the developing mushroom, resulting in increased productivity up to 30% with a shorten cultivation cycle^15^.

Ruffling is a process whereby the *Agaricus* mycelium which is growing into the casing is thorough mixed through the whole casing depth. This practice allows for more accurate control of the first flush and uniformity of production due the diffusion of CO_2_ and the admission of O_2_ when the growing room is aerated^16^. Recommendations for the initiation of fructification and the temperatures used for the Sun mushroom vary.

Some authors recommend the reduction of the temperature to approximately 20 °C for primordia induction while others maintain the temperature constant at 23 °C and 24–26 °C^12,17–19^. Primordia initiation and harvest intervals are temperature sensitive. Its management is fundamental for the timing of the flushes for harvest. In this manuscript we report production and crop responses from nutritional supplementation of compost at spawning, ruffling of the casing layer, and various temperature management schema for induction of primordia in the medicinal mushroom *A. subrufescens*.

## Materials and methods

### Experimental design

The experimental factorial design was a 4×2×2 with six replicates completely randomized. Factor 1 had four kinds of supplements (three commercial supplements and a non-supplemented control). For Factor 2 the casing was either ruffled or not. Factor 3 used two mushroom production chambers to achieve two schemes of primordia induction conditions. Ninety-six small trays (0.145 m^2^ surface area) were randomized, equally divided between two identical growth chambers and arranged on two levels.

### Spawn

*A. subrufescens* strain ABL 99/30 (Centro de Estudos em Cogumelos, FCAT-UNESP, Brazil) was selected for the experiment. This strain, collected in Piedade (São Paulo, Brazil) in 1999, is characterized by its medium to small size fruit bodies, strong texture, high yield and precociousness, reduced time to first harvest, and slightly lower fructification temperature^20^. Spawn was prepared according to Andrade *et al*.^21^ in these steps: production of subculture from culture bank, production of mother spawn, and production of grain spawn for compost inoculation. Compost was inoculated with grain spawn at a rate of 15 g kg^−1^ of fresh weight of compost.

### Substrates and supplements used

Commercial Phase II compost used for production of *A. bisporus* that was based on wheat straw and chicken manure (Compost Villacasa S.L., Casasimarro, Cuenca, Spain) was used as substrate for the Sun mushroom production. Along with the non-supplemented compost, three commercial products with high protein content were evaluated as nutritional supplements for mushroom cultivation: Promycel^®^ 600 (Amycel Europe, Vendôme, France), Champfood^®^ (ChampFood International, Vierlingsbeek, The Netherlands) and Calprozime^®^ (Calliope, Breziers, France). The physical, chemical and biological characteristics of the compost and supplements were determined according to Zied *et al*.^22^ (Table 1).

**Table 1:** Chemical characteristics of compost and supplements.

Characteristics	Compost	Promycel 600	Champfood	Calprozime
pH (1:5, w/v)	7.54	5.82	6.19	8.95
Moisture (g kg^−1^)	684.0	119.0	144.2	109.7
Total nitrogen (g kg^−1^)	20.5	82.1	82.7	64.8
Protein (g kg^−1^)	128.1	513.0	516.6	405.1
Ash (g kg^−1^)	273.6	64.3	65.1	117.5
Organic matter (g kg^−1^)	726.5	935.7	934.9	882.5
C/N	20.6	6.6	6.6	7.9
Crude fibre (g kg^−1^)	276.8	97.7	65.5	144.9
Crude fat (g kg^−1^)	3.9	31.2	10.0	16.5
N-free extract (g kg^−1^)	317.6	402.3	342.8	316.1
Hemicellulose (g kg^−1^)	178.1	188.7	342.2	227.9
Cellulose (g kg^−1^)	131.3	50.0	60.1	95.0
Lignin (g kg^−1^)	268.9	132.3	91.3	123.3
Neutral-det. sol. (g kg^−1^)	148.3	564.6	441.3	436.4

Sun mushroom trials were carried out in two 20 m^3^ experimental growth chambers equipped with automatic control of temperature, relative humidity and carbon dioxide. At spawning, the compost was thoroughly incorporated with 10 g kg^−1^ of Promycel 600, Champfood or Calprozime. Trays were cased 13 days after spawning with a peat-based commercial mixture, Euroveen^®^ (Euroveen BV, Grubbenvorst, The Netherlands) to the depth of 4 cm.

The casing material of one-half of the trays was deeply ruffled by-hand when the mycelia appeared on the surface 8 days after casing application. A day later, the environmental temperature, relative humidity, and carbon dioxide level were decreased to induce primordia formation.

**Figure 1:**
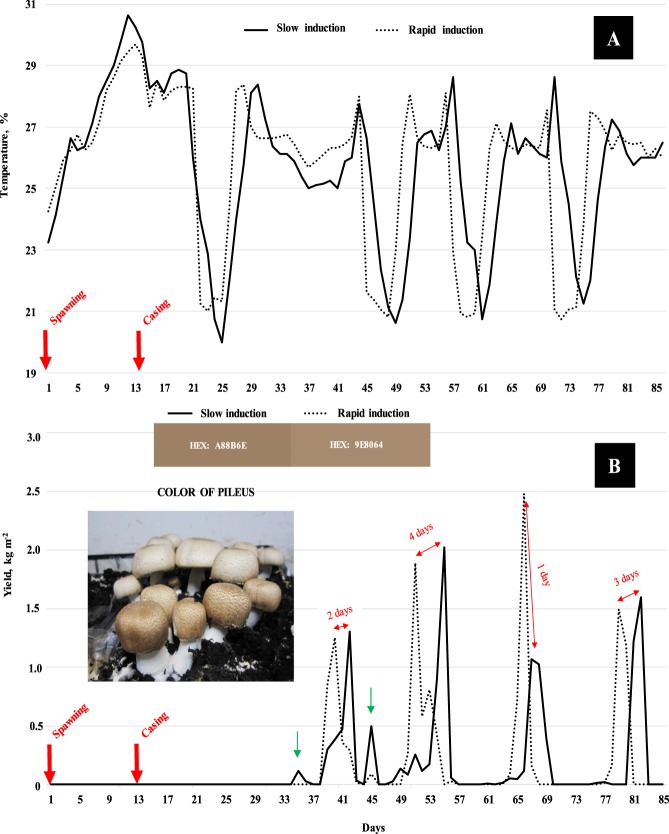
(**A**) Temperature management used for slow and rapid primordia induction; (**B**) Yield obtained by flushes with slow and rapid induction of primordia (Factor 3). The picture demonstrates differences in pileus color (HEX value represents an international color code obtained by the measure of L *, a * and b *, https://www.nixsensor.com/free-color-converter/). Value of slow induction (L* 56.43, a* 8.55 and b* 19.74) and rapid induction (L* 59.56, a* 8.05 and b* 19.92). Production rate of slow induction was 0.67 kg dt^−1^ d^−1^ and rapid induction was 0.73 kg dt^−1^ d^−1^.

Two fruiting induction methods were evaluated. Initially, the compost temperatures were maintained at 28 °C from spawning through the day after ruffling. We followed the strategy used for *A. bisporus* that the compost temperature is needed to be lowered 7-9 °C below the optimal mycelial growth temperature in order to initiate primoridia^23^. Thus, we reduced the compost temperature from 28 to 20 °C in two schemes (Fig. 1A). Slow induction of primordia was managed by reducing the compost temperature by 2 °C d^−1^ until 20 °C was reached. The compost temperature was maintained at 20 °C for two days. After which it was increased by 2 °C d^−1^ until 28 °C was achieved (day 29)^24^. Rapid induction of primordia was accomplished by reducing the temperature to 20 °C in a day^25^. It was kept at this temperature for 4 days and then increased to 28 °C in one day (day 26). Drop air temperature to 20 °C for fruiting and compost temperature of 26 °C and 700 ppm CO_2_ during the harvest were used in both cases. The total growth cycles lasted 85 days and four flushes of mushrooms were obtained.

### Production parameters

Mushrooms were harvested daily at their optimal commercial development stage prior to opening of the veil. The measured or calculated agronomic behavior (earliness, biological efficiency, production rate, number of mushrooms, unitary weight and yield per area) were assessed on untrimmed mushrooms. Whereas, proximate analysis (dry matter content of harvested mushrooms, protein, fat, carbohydrate and fiber) were evaluated on trimmed mushrooms of the peak harvest day of first flush. Earliness, or days to the first harvest, was expressed as the number of days between casing and the beginning of the first flush harvest. Biological efficiency, an estimation of mushrooms’ ability to convert substrate into fruiting bodies, was calculated by dividing the total fresh weight of mushrooms harvested from the whole crop over all the flushes by the total substrate dry weight, being expressed as kg dt^−1^ compost. The size of the mushrooms, expressed as unit weight in g, was calculated from the yield and number of mushrooms harvested. Yield is expressed as kg per cultivated area. The production rate is based on the total biological efficiency and the time elapsed from the entry of the substrate into the growth chambers to the last day of harvest.

### Proximate analysis and dry matter

On the day when most mushrooms were harvested during the first flush, the proximate analysis and dry matter were determined for those mushrooms of uniform size and at the same development stage according to Zied *et al*.^22^. Dry matter were determined by measuring the loss of weight after oven drying at 105 °C for at least 72 h or until stable loss was recorded. Mushroom protein was calculated by multiplying the total nitrogen by a factor of 4.38^26^. Crude fat was estimated gravimetrically by filter bag technique^27^. Total carbohydrate content was calculated by subtracting the sum of the crude protein, total fat, water and ash from the total weight of the mushrooms. The crude fiber content utilized the Weende technique adapted to the filter bag technique^28^. For ash content carpophores were ashed at 540 °C for at least 6 h^29^. The surface colour of mushrooms was measured by reflectance using a Minolta chroma meter CR-300 colorimeter previously calibrated with a calibration plate CR-A43 (L* = 96.12, a*=−0.11, b*=+2.66) and illuminant D65 as a light source.

### Statistical analyses

All data (measured or calculated) were analyzed using ANOVA (Statistical Graphics Corp., Princeton, NJ, USA) and the Tukey-HSD test employed to separate means (P = 0.05). Sigma Stat 3.5 software was used to generate Pearson Product Moment correlations between the values for earliness, biological efficiency, number and weight of mushrooms with the chemical characteristics of the supplements.

## Results and discussion

### Factor 1 (compost supplementation)

Supplementation of compost at spawning significantly increased yield, biological efficiency and individual mushroom weight, as well as decreasing earliness to first harvest (Table 2) regardless of disturbing (ruffling) the mycelia in the casing layer or speed of primordia initiation.

**Table 2:** Agronomic behavior of non-supplemented and supplemented compost (Factor 1).

Treatment	Earliness (days from casing)	Biological efficiency (kg dt^−1^ compost)	Number of mushroom (m^−2^)	Weight of mushroom (g)
Non-supplemented	28.7 b	53.71 ab	533	23.0 b
Promycel 600	27.3 a	52.75 b	464	26.5 a
Champfood	27.2 a	60.65 a	558	25.9 ab
Calprozime	27.6 a	58.18 ab	555	24.6 ab
Mean	27.7	56.33	528	25.0

For each factor within a column, values followed by a different letter are significantly different at 5% level according to Tukey’s HSD test. The absence of letters in the column indicates non-significance.

**Figure 2:**
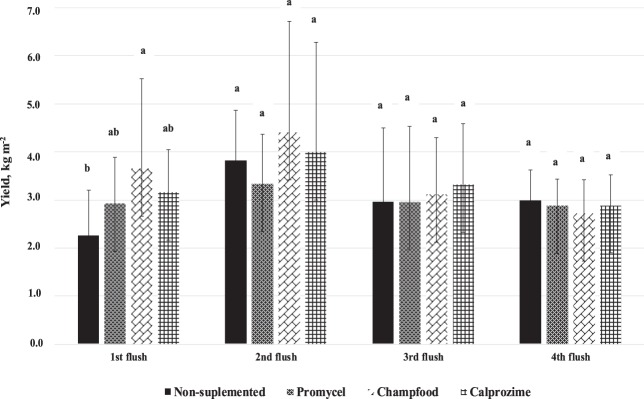
Yield obtained by flushes with non-supplemented and supplemented compost (Factor 1). For each flush, values followed by a different letter are significantly different at 5% level according to Tukey’s HSD test.

Increased production from supplementation is the commercially desirable outcome. Total production over four harvest flushes showed a significant increase in yield by Champfood over any other treatment. The gain in production was realized primarily in the first flush (Fig. 2). The other flushes did not demonstrate significant yield responses for supplementation. Rankings among treatments for progressive accumulated yield (data not shown) was consistent throughout the trial. The positive effect of supplementation on yield (up to 50%) of the Sun mushroom has been reported by other authors as well. They have noted that the positive response of supplementation is also mushroom strain related^30^. In a study specifically regarding the economic aspects of compost supplementation for *Agaricus bisporus* production, Randle and Smith^31^ estimated that the cost of supplementation is covered by a yield increase of 1.5 kg m^−2^. In our study, untrimmed yields over four flushes were non-supplement (12.01 kg m^−2^), Promycel (12.11 kg m^−2^), Calprozime (13.36 kg m^−2^) and Champfood (13.91 kg m^−2^). In our study, the only supplement that would be economically viable would be the Champfood.

**Figure 3:**
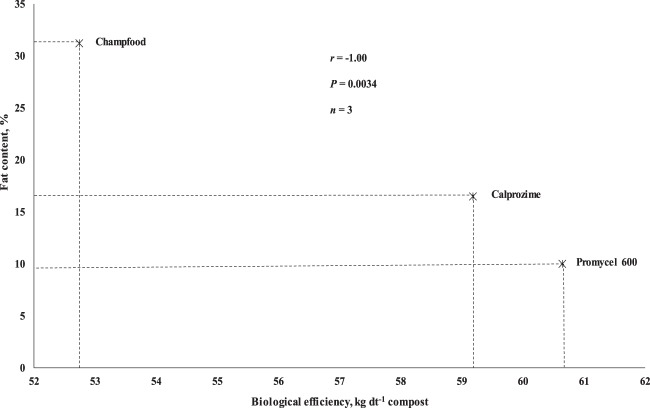
Correlations between biological efficiency and the contents of fat.

An interesting question is “which characteristic of the supplement influences production”? Several authors have indicated that the supplement used in the production of *Agaricus* should be rich in N (vegetable protein)^32,33^. Others have noted that the supplement should have low levels of P, K and Mg^30^. The supplements evaluated varied substantially among their chemical characteristics. Promycel 600 and Champfood supplements were about 21% higher in total nitrogen than Calprozime. However, Promycel 600 was lower in hemicellulose and cellulose but higher in lignin. Champfood was lower in crude fat and Calprozime higher in pH, ash and fiber (Table 1). In our study we observed a negative correlation between biological efficiency and fat content of the supplement (r = −1.00 and P = 0.0034) (Fig. 3). In this sense, we suggest that future experiments be carried out using nutritional supplements with high protein and low fat content, chemical characteristic similar to that observed by Champfood.

Finally, the commercial *A. bisporus* compost used in the present research was poor, with values of 2.05% N content and C/N ratio of 20.6/1. Some of the literature report higher N values such as N – 2.6 and lower C/N ratio – 16/1^34^. *A. subrufescens* is highly sensitive to ammonia in compost, and when the initial concentration of nitrogen is high, there is a more intense production of ammonia, depending on the degradation of protein^35^. Thus, organic supplements rich in N can improve the quality of the compost, avoiding the excessive application of mineral N during the composting phase I, which can reduce the losses of ammonia volatization.

Supplementation significantly decreased the time (earliness) to first harvest. All the supplements evaluated significantly reduced this time by over one day (Table 2). Although all supplements decreased the time to first harvest, only Champfood demonstrated a significant increase in yield over all other treatments (Fig. 2; Table 2).

Earliness is an important parameter in improving the mushroom technology. Lower earliness values were observed in the cultivation of *A. bisporus*, ranging from 19.0 to 21.7 days, and the addition of supplements in the compost had no significant influence on earliness^36,37^, different from the results obtained in this manuscript which the supplementation had significant influence on earliness. Dias *et al*.^38^ (as ourselves, Table 2) observed that overall production in *A. subrufescens* was greater when the time to harvest (earliness) was shorter.

Compost supplementation increased individual mushroom weight, however, only Promycel was significant (Table 2). Greater individual mushroom weight is extremely important for the Sun mushroom since the product is valued for its larger size, washed by-hand and dehydrated. A larger mushroom makes the handling process more efficient. The strain used in this study (ABL 99/30) strain is characterized as a smaller one^20^ which can be improved through addition of a compost supplement at spawning.

### Factor 2 (ruffling technique)

**Figure 4:**
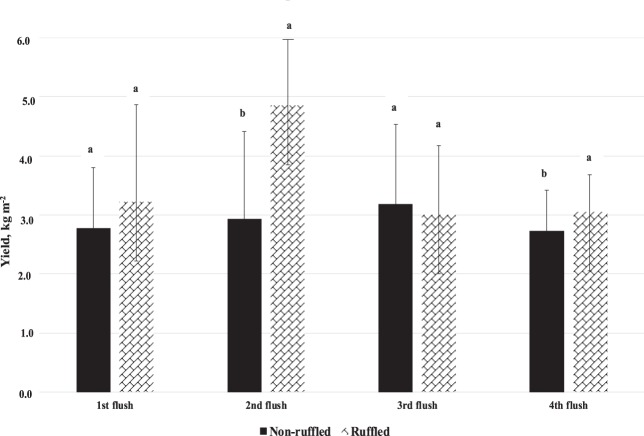
Yield obtained by flushes with non-ruffled and ruffled casing layer (Factor 2). For each flush, values followed by a different letter are significantly different at 5% level according to Tukey’s HSD test.

The ruffling technique significantly improved yield, biological efficiency and number of total mushrooms (Table 3). Biological efficiency was greater by 21%. This gain in productivity came from significantly higher yields on the second and fourth flushes (Fig. 4). This fact occurs naturally in the cultivation of *A. subrufescens*, specifically, when a flush (2nd) present good yield, the subsequent one (3rd) tends to present less yield. The importance of the ruffling technique becomes essential to leave the casing layer with a lower mycelium density, which allows a better gas exchange until the end of the harvest period (4th flush). Despite the significant increase in total mushrooms through ruffling the casing layer, the individual mushroom weight was not significantly influenced by the ruffling technique.

**Table 3:** Agronomic behavior of non-ruffled and ruffled casing layer (Factor 2).

Treatment	Earliness (days from casing)	Biological efficiency (kg dt^−1^ compost)	Number of mushroom (m^−2^)	Weight of mushroom (g)
Non-ruffled	27.7	50.99 b	491 b	24.7
Ruffled	27.7	61.66 a	564 a	25.3
Mean	27.7	56.33	528	25.0

For each factor within a column, values followed by a different letter are significantly different at 5% level according to Tukey’s HSD test. The absence of letters in the column indicates non-significance.

Dias *et al*.^38^, after ruffling three casing materials (sphagnum peat moss, loam soil, coconut coir), observed that a loam soil casing was highly superior to either peat or coir. Soils typically have a more dense compact structure than peat or coir. In contrast, in the cultivation of *A. bisporus* loam soil was used early in its commercial development. However, in more recent years materials or blends (such as certain peat mosses and sugar beet lime) that provide low density, good water holding capacity and yet an open texture to reduce buildup of carbon dioxide have been used^16,39,40^.

The Sun mushroom has a denser mycelium than *A. bisporus* and produces a greater formation of stroma in its 65 to 120 day cultivation cycle^12,19,41,42^. Our casing material, used commercial in The Netherlands for *A. bisporus* production, had an open structure, a high porosity (93%), a high water holding capacity (4.92 kg kg^−1^) and a low bulk density (0.654 g cm^−3^).

The ruffling technique may increase the cost of production, especially if hand labor is required. Ruffling machines have been used on *A. bisporus* shelf farms for decades^16^. If *A. subrufescens* would be grown on a shelf system, little, if any, adaptations would be necessary.

### Factor 3 (temperature management)

After spawning, the temperature of the compost in both chambers (slow and rapid induction) ranged from 23 ± 1 °C to approximately 30 ± 0.5 °C (Fig. 1A). Heating of the compost results from the metabolic process of the mycelian colonization of the substrate with its release of CO_2_ and heat. After casing, the compost temperatures were maintained at 28 ± 0.5 °C for 7 days. On day 21 the casing layer was ruffled (8 days after casing) and the temperature reduction of the compost began in both chambers. Slow induction of primordia was accomplished by day 29. Whereas, the rapid initiation of primordia was complete on day 26.

Harvesting of first flush started for both chambers on day 39 and ended on day 43. Maximum production in the rapid induction occurred 2 days before the slow (Fig. 4B). Pre- and post-first flush occurred in the slow induction, outside the bulk harvest days 39-43 (green arrows in Fig. 1B). Depending on the quality of these mushrooms, this pre- and post- occurrence may not be undesirable.

In the second flush, the duration of the slow induction harvest continued for 9 days and the rapid induction for two days less. The maximum production of rapid induction occurred 4 days prior to the slow induction.

In the third flush, the duration of slow induction harvest lasted 7 days and the rapid induction lasted 6 days. The highest yield harvested in any single day in the crop occurred in this flush with the amount of 2.484 kg of mushrooms per m^2^ for the rapid induction method. The yield difference between primordia induction methods in this flush (3.47 kg m^−2^, rapid induction; and 2.72 kg m^−2^, slow induction) was the only significance during the experiment.

Finally, the last flush terminated 3 days earlier for the rapid induction. However, both methods were harvest for 4 days. Additional the production rate was significant higher in the rapid induction method (0.73 kg dt^−1^ d^−1^) than in the slow induction method (0.67 kg dt^−1^ d^−1^) (data not shown).

The speed of induction significantly affected the color of the mushroom. Primordia induction slowly had a significantly darker pileus color (L* - 56.43, a* - 8.55 and b* 19.74). The color of the pileus is an indicator of the maturity of Sun mushroom. The color of the pileus lightens in color as the veil is closer to opening.

The biological efficiency was not significantly affected by the mushroom induction temperature method. Our biological efficiencies (57.84%, rapid induction; and 54.81%, slow induction) were better than other reports that managed the temperatures of primordia initiation differently. Martos *et al*.^43^ obtained a biological efficiency of 40.0, 38.0 and 39.6% when the temperature was lowered to 16 °C and then maintained for 4, 6 and 8 days, respectively. Wang *et al*.^19^ maintained a constant temperature between 24 and 26 °C with the highest biological efficiency being 41.4% over a cultivation period of 120 days of 6 flushes.

### Proximate analysis

In general, the composition of mushrooms is water (90%), protein (2–40%), fat (2–8%), carbohydrates (1–55%), fiber (3–32%) and ash (8–10%)^44^. The composition of *A. subrufescens* presents low water content, crude fat, crude fiber and ash, while high protein content, total carbohydrate and mean values of available carbohydrates and energy value^45^. Composition reported in this paper is, in common, of the same magnitude as those reported in the literature^35,46,47^.

The mushrooms were not significantly affected by supplementation, or presence or absence of ruffling of the casing layer. Speed of induction did significantly affect the fat content. All other parameters were not influenced (Table 4). Siqueira *et al*.^35^ evaluating composts prepared with different initial concentrations of nitrogen, found that the chemical analysis of dry mushrooms revealed not significant differences in protein, fat and ash content between the treatments, while significant differences in fiber (source of b-glucan) content were observed.Supplementation of A. bisporus compost, on-the-other-hand, increased the protein, fiber and ash content and reduced the carbohydrate content of the harvested mushrooms^15^. The study of Eira *et al.*^46^ reported the influence of different strains and morphogenetic stages on the proximate analysis of A. subrufescens.

**Table 4:** Chemical characteristics of the mushrooms due to the supplementation of the compost (Factor 1), the used of the ruffling technique (Factor 2) and the temperature management in the induction of primordia (Factor 3).

Treatment (Factors)	Dry matter (%)	Protein (Nx4.38, g kg^−1^)	Fat (g kg^−1^)	Carbohydrates (g kg^−1^)	Fiber (g kg^−1^)	Ash (g kg^−1^)
Non-supplemented	12.47	283.6	15.5	632.1	62.3	68.8
Promycel 600	12.39	272.6	17.2	642.0	62.7	68.2
Champfood	12.48	277.3	17.6	637.4	61.8	67.7
Calprozime	12.37	273.2	17.0	642.3	64.6	67.5
Non-ruffled	12.49	278.8	16.5	637.0	61.7	67.7
Ruffled	12.36	274.5	17.1	640.0	64.0	68.4
Rapid induction	12.33	272.5	18.6 a	640.9	63.6	68.0
Slow induction	12.52	280.9	15.0 b	636.0	62.1	68.1
Mean	12.43	276.7	16.8	638.5	62.8	68.0

For each factor within a column, values followed by a different letter are significantly different at 5% level according to Tukey’s HSD test. The absence of letters in the column indicates non-significance.

## Conclusions

Champfood supplement promotes a reduction in the value of earliness and an increase of 1^st^ flush yield. The ruffling technique provided an increase in biological efficiency due to the great number of mushrooms harvested. Rapid primordia induction allowed the crop cycle to end 3 days earlier than the slow primordia induction, providing a higher production rate.

## Supplementary information

Supplementary information.
