# Supplementary material for: Optimization of cultivation techniques improves the agronomic behavior of Agaricus subrufescens
Source: Sci Rep. 2020 May 18;10:8154. doi: 10.1038/s41598-020-65081-2 (PMC7235075; doi:10.1038/s41598-020-65081-2)

**SUPPLEMENTARY MATERIAL**

**Optimization of cultivation techniques improves the agronomic behavior of *Agaricus subrufescens***

Arturo Pardo-Giménez^1^, José Emilio Pardo^2^, Eustáquio Souza Dias^3^, Danny Lee Rinker^4^, Cinthia Elen Cardoso Caitano^5^ & Diego Cunha Zied^5*^

^1^Centro de Investigación, Experimentación y Servicios del Champiñón (CIES), Quintanar del Rey, Spain. ^2^Escuela Técnica Superior de Ingenieros Agrónomos, Universidad de Castilla-La Mancha, Albacete, Spain. ^3^Universidade Federal de Lavras, Departamento de Biologia, Lavras, Brazil.

^4^University of Guelph, Vineland Campus, VinelandStation, Guelph, Canada. ^5^Universidade Estadual Paulista (UNESP), Faculdade de Ciências Agrárias e Tecnológicas (FCAT), Dracena, Brazil.

*Address correspondence to Diego Cunha Zied, [dczied@gmail.com](mailto:dczied@gmail.com) Rod. Cmte João Ribeiro de Barros, km 651, Bairro das Antes, 17900-000, Dracena, SP, Brazil. Fax: + 55 18 3821-8208.

**ORCID:** 0000-0003-2279-4158

Evaluating the interaction of the three factors, it was observed that only the number of mushrooms and the unitary weight were not significantly influenced (*p* ≤ 0.05) on the agronomic behavior, which made it very difficult to present the results obtained. In this sense the unfolding of the 16 treatments are presented in Table S1. The higher biological efficiency was obtained with Champfood supplement/ruffled technique/rapid induction, on the other hand lower biological efficiency were obtained with non-supplemented compost/non-ruffled/rapid induction and Promycel/non-ruffled/slow induction. Different form the results obtained for the agronomic parameters, the chemical characteristics of mushroom do not show significantly difference for the various treatments (Table S2).

**Table S1** The results obtained for the agronomic behavior assessed in the mushrooms originating from the various treatments

|  | Number of mushrooms  m^-2^ | Unitary weight  (g) | Yield (kg m^-2^) | | | | | | Biological efficiency  (kg dt^-1^ compost) | Production rate  (kg dt^-1^ d^-1^) | Earliness  (days from casing) |
| --- | --- | --- | --- | --- | --- | --- | --- | --- | --- | --- | --- |
|  |  |  | 1st  flush | 2nd  flush | 3rd  flush | 4th  flush | Total | Total net yield |  |  |  |
| NS/R/RI | 568 | 23.5 | 1.80 b | 4.62 ab | 4.14 | 2.52 ab | 13.08 abc | 11.99 ab | 58.53 ab | 0.73 abcd | 27.9 ab |
| NS/NR/RI | 483 | 22.6 | 1.94 b | 3.20 ab | 2.58 | 2.88 ab | 10.58 c | 9.73 b | 47.33 b | 0.60 d | 29.0 ab |
| PRO/R/RI | 475 | 27.4 | 2.59 ab | 4.23 ab | 2.50 | 3.58 a | 12.91 abc | 11.97 ab | 56.25 ab | 0.71 abcd | 27.4 ab |
| PRO/NR/RI | 491 | 28.2 | 3.44 ab | 3.20 ab | 3.91 | 2.96 ab | 13.51 abc | 12.69 ab | 58.87 ab | 0.74 abcd | 27.0 ab |
| CHF/R/RI | 614 | 26.2 | 4.11 ab | 5.51 a | 3.64 | 2.96 ab | 16.21 a | 14.81 a | 70.67 a | 0.89 a | 26.8 ab |
| CHF/NR/RI | 460 | 26.3 | 2.90 ab | 3.28 ab | 3.22 | 2.22 b | 11.62 abc | 10.68 ab | 50.63 ab | 0.64 abcd | 27.0 ab |
| CPZ/R/RI | 622 | 25.7 | 3.32 ab | 6.23 a | 3.39 | 2.99 ab | 15.91 ab | 14.58 a | 69.37 a | 0.87 ab | 26.6 b |
| CPZ/NR/RI | 509 | 24.8 | 2.83 ab | 1.69 b | 4.35 | 2.87 ab | 11.72 abc | 10.87 ab | 51.07ab | 0.64 abcd | 27.3 ab |
| NS/R/SI | 581 | 22.5 | 2.90 ab | 4.18 ab | 2.62 | 3.28 ab | 12.98 abc | 11.84 ab | 58.05 ab | 0.71 abcd | 29.1 a |
| NS/NR/SI | 501 | 23.2 | 2.37 b | 3.28 ab | 2.52 | 3.23 ab | 11.39 bc | 10.70 ab | 50.93 ab | 0.62bcd | 28.9 ab |
| PRO/R/SI | 469 | 24.5 | 2.66 ab | 4.07 ab | 2.10 | 2.64 ab | 11.47 abc | 10.64 ab | 49.98 ab | 0.61 cd | 27.6 ab |
| PRO/NR/SI | 420 | 26.1 | 3.05 ab | 1.84 b | 3.32 | 2.34 ab | 10.54 c | 9.83 b | 45.92 b | 0.56 d | 27.5 ab |
| CHF/R/SI | 634 | 27.0 | 5.07 a | 5.46 a | 2.80 | 2.68 ab | 16.01 ab | 14.77 a | 69.80 a | 0.86 abc | 28.2 ab |
| CHF/NR/SI | 525 | 23.9 | 2.55 ab | 3.40 ab | 2.82 | 3.04 ab | 11.81 abc | 11.00 ab | 51.48 ab | 0.63 abcd | 26.8 ab |
| CPZ/R/SI | 554 | 25.4 | 3.32 ab | 4.46 ab | 2.80 | 3.35 ab | 13.91 abc | 12.66 ab | 60.60 ab | 0.74 abcd | 28.2 ab |
| CPZ/NR/SI | 537 | 22.5 | 3.18 ab | 3.59 ab | 2.75 | 2.35 ab | 11.88 abc | 10.93 ab | 51.72 ab | 0.63 abcd | 28.5 ab |
| Mean | 528 | 25.0 | 3.00 | 3.89 | 3.09 | 2.87 | 12.85 | 11.86 | 56.33 | 0.70 | 27.7 |

Values followed by a different letter within a column are significantly different at 5% level according to Tukey’s HSD test.

NS: non-supplemented; PRO: Promycel; CHF: Champfood; CPZ: Calprozime; R: ruffled; NR: non-ruffled; RI: rapid induction; SI: slow induction.

**Table S2** Chemical analyses of mushrooms originating from the various treatments

|  | Water  (g kg^-1^) | Crude protein  (Nx4.38, g kg^-1^ d.m.) | Crude fat  (g kg^-1^ d.m.) | Total carbohydrates  (g kg^-1^ d.m.) | Nitrogen free extract  (g kg^-1^ d.m.) | Crude fiber  (g kg^-1^ d.m.) | Ash  (g kg^-1^ d.m.) | Energy value (kcal/100g d.m.) |
| --- | --- | --- | --- | --- | --- | --- | --- | --- |
|  |  |  |  |  |  |  |  |  |
| NS/R/RI | 874.5 | 274.9 | 14.7 | 642.8 | 576.9 | 66.0 | 67.6 | 357 |
| NS/NR/RI | 871.0 | 292.8 | 16.3 | 623.9 | 565.7 | 58.2 | 67.0 | 361 |
| PRO/R/RI | 877.8 | 275.1 | 19.0 | 636.6 | 575.1 | 61.5 | 69.4 | 360 |
| PRO/NR/RI | 881.6 | 267.0 | 18.2 | 645.7 | 580.5 | 65.7 | 69.1 | 359 |
| CHF/R/RI | 879.4 | 261.8 | 21.0 | 648.6 | 587.3 | 61.3 | 68.6 | 362 |
| CHF/NR/RI | 876.9 | 258.2 | 20.1 | 655.3 | 594.2 | 61.1 | 66.4 | 363 |
| CPZ/R/RI | 878.2 | 271.4 | 21.9 | 638.4 | 568.5 | 69.8 | 68.3 | 359 |
| CPZ/NR/RI | 874.1 | 278.7 | 17.5 | 636.3 | 570.9 | 65.4 | 67.6 | 359 |
| NS/R/SI | 878.0 | 291.6 | 15.2 | 622.8 | 559.2 | 63.6 | 70.5 | 357 |
| NS/NR/SI | 877.9 | 275.2 | 15.7 | 639.1 | 577.7 | 61.5 | 70.0 | 359 |
| PRO/R/SI | 870.9 | 256.7 | 15.7 | 662.3 | 600.5 | 61.8 | 65.3 | 361 |
| PRO/NR/SI | 874.2 | 291.5 | 15.8 | 623.6 | 562.0 | 61.7 | 69.1 | 358 |
| CHF/R/SI | 874.6 | 287.3 | 15.0 | 628.3 | 566.2 | 62.1 | 69.4 | 358 |
| CHF/NR/SI | 870.0 | 301.9 | 14.5 | 617.4 | 554.6 | 62.8 | 66.3 | 358 |
| CPZ/R/SI | 877.5 | 277.2 | 14.6 | 640.3 | 574.5 | 65.8 | 67.9 | 357 |
| CPZ/NR/SI | 875.3 | 265.4 | 13.9 | 654.5 | 597.2 | 57.3 | 66.3 | 362 |
| Mean | 875.7 | 276.7 | 16.8 | 638.5 | 575.6 | 62.8 | 68.0 | 359 |

Values followed by a different letter within a column are significantly different at 5% level according to Tukey’s HSD test.

d.m.= dry matter; NS: non-supplemented; PRO: Promycel; CHF: Champfood; CPZ: Calprozime; R: ruffled; NR: non-ruffled; RI: rapid induction; SI: slow induction


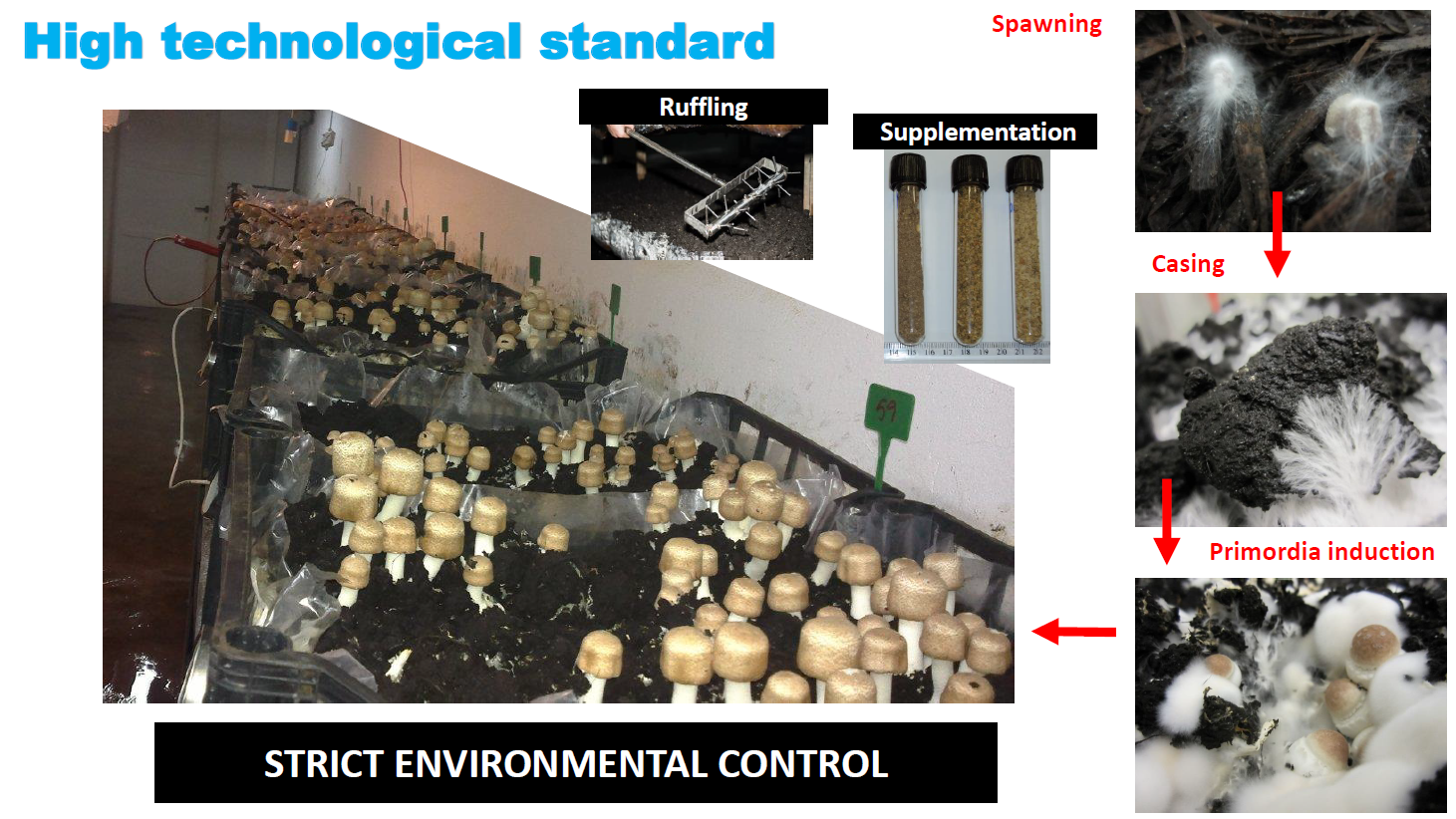

Supplement: Supplementary file 1 — Supplementary information. [file 41598_2020_65081_MOESM1_ESM.docx]
